# Laparoscopic Transabdominal Cerclage Despite the Absence of Prior Pregnancy Loss: A Case of Profound Acquired Cervical Anatomical Defects After Repeated Conization

**DOI:** 10.1111/jog.70456

**Published:** 2026-08-10

**Authors:** Kensuke Suzuki, Takayuki Iriyama, Gentaro Izumi, Osamu Hiraike, Miyuki Harada, Yasushi Hirota

**Affiliations:** ^1^ Department of Obstetrics and Gynecology, Faculty of Medicine The University of Tokyo Tokyo Japan

**Keywords:** cervical conization, cervical stenosis, cervicoisthmic cerclage, laparoscopic transabdominal cerclage, pregnancy

## Abstract

Repeated cervical conization can result in cervical shortening and substantial structural and functional impairment. A 35‐year‐old woman developed cervical stenosis and hematometra after undergoing two conizations. The vaginal cervix and external os were unidentifiable, drainage was unsuccessful, and laparoscopy‐assisted recanalization proved technically challenging as multiple false passages formed before achieving uterine access. Repeated dilation maintained cervical patency, and intrauterine insemination achieved pregnancy after cervical factor infertility was diagnosed. At 9 weeks of gestation, the residual endocervical glandular region measured 11.9 mm, and the vaginal cervix was nearly absent. As the cumulative clinical course demonstrated severe anatomical disruption that precluded transvaginal cerclage, laparoscopic transabdominal cervicoisthmic cerclage was performed at 13 weeks and 3 days of gestation. Uterine artery pulsatility indices remained within the reference range. A 2114‐g infant was delivered by cesarean section at 37 weeks and 5 days. Severe anatomical cervical defects may warrant transabdominal cerclage beyond conventional history‐based indications.

## 1 Introduction

Cervical cerclage is most commonly performed using a transvaginal approach. Transabdominal cerclage is generally reserved for women with recurrent pregnancy loss or preterm birth despite previous transvaginal cerclage or when cervical anatomy precludes a transvaginal procedure. Recently, laparoscopic transabdominal cerclage has been increasingly adopted [1].

Repeated cervical conization can result in cervical shortening, tissue loss, distortion, and stenosis. Although pregnancy after conization is associated with an increased risk of miscarriage and preterm birth [2, 3, 4], prophylactic cerclage based solely on a history of conization has not been established. However, the severity of post‐conization cervical injury exists along a spectrum. Repeated excisions may lead not only to cervical shortening but also to profound anatomical and functional disruption, including near‐complete loss of the vaginal cervix, obliteration of the external cervical os, hematometra, and loss of an accessible cervical canal.

Indications for transabdominal cerclage in women without conventional history‐based criteria but with severe acquired cervical anatomical defects remain poorly defined. This case report describes a patient whose cumulative clinical course after repeated conizations resulted in profound cervical disruption, rendering transvaginal cerclage technically unfeasible and necessitating laparoscopic transabdominal cervicoisthmic cerclage during pregnancy.

## 2 Case Report

A 35‐year‐old woman had undergone cervical conization for cervical intraepithelial neoplasia grade 3 (CIN3) 8 years earlier, with negative resection margins. Recurrent CIN3 was diagnosed 1 year later, and a second conization was performed. Two years thereafter, she was referred to our hospital with hematometra secondary to cervical stenosis.

**FIGURE 1:**
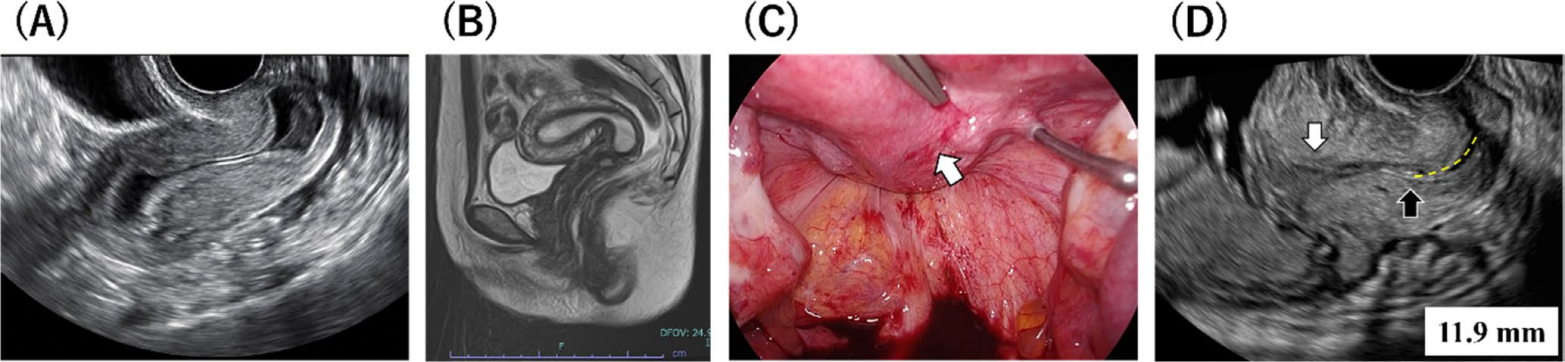
Imaging findings of hematometra, laparoscopic cervical recanalization, and cervical morphology at 9 weeks of gestation. (A) Transvaginal ultrasonography demonstrating blood accumulation within the uterine cavity and cervical canal. (B) Sagittal T2‐weighted magnetic resonance image demonstrating marked distension of the uterine cavity and cervical canal caused by severe cervical stenosis. (C) Laparoscopy‐assisted recanalization. The original cervical canal was difficult to identify despite laparoscopic guidance, and multiple false passages were created during the initial recanalization attempts. The uterine cavity was ultimately accessed using a central venous catheterization kit, followed by cervical reconstruction and dilation. The white arrow indicates the actual catheter placement site. (D) Transvaginal ultrasonography at 9 weeks of gestation showing a residual endocervical glandular region measuring 11.9 mm from the external cervical os to its cranial end (black arrow). The white arrow indicates the anatomical internal cervical os.

At presentation, neither the vaginal cervix nor the external cervical os was identifiable on speculum examination. Transvaginal ultrasonography and magnetic resonance imaging demonstrated marked hematometra extending from the uterine cavity into the cervical canal, consistent with functionally cervical outflow obstruction (Figure 1A,B).

Transvaginal drainage under sedation was unsuccessful, as neither the external cervical os nor the cervical canal could be reliably identified. Therefore, laparoscopy‐assisted transvaginal cervical recanalization was performed. Even under laparoscopic guidance, identification of the original cervical canal was technically challenging, and multiple false passages were created during the initial attempts. The uterine cavity was subsequently accessed using a central venous catheterization kit, followed by cervical reconstruction and dilation (Figure 1C). Thereafter, cervical patency and menstrual drainage were maintained with repeated Hegar dilation at 1‐ to 2‐month intervals.

When the patient desired pregnancy, conception was achieved by intrauterine insemination following cervical reconstruction and repeated cervical dilation. The preceding cervical stenosis and difficulty accessing the cervical canal were considered to be likely contributors to infertility.

Transvaginal ultrasonography at 9 weeks of gestation showed that the distance from the external cervical os to the cranial end of the residual endocervical glandular region was 11.9 mm (Figure 1D), with the anatomical internal cervical os located further cranially. The vaginal cervix was almost completely absent.

The decision to perform cerclage was based not on the 11.9‐mm measurement alone but on cumulative evidence of profound cervical disruption, including near‐complete loss of the vaginal cervix, an unidentifiable external os, hematometra, failed transvaginal drainage, technically challenging recanalization with multiple false passages, and dependence on repeated dilation.

Because the cervix could not be adequately exposed or mobilized through the vaginal route, transvaginal cerclage was considered technically unfeasible. Although the patient had no history of miscarriage or preterm birth, concern remained that the profoundly disrupted residual cervix could not provide adequate mechanical support throughout pregnancy. The patient received detailed counseling regarding expectant management and alternative treatment options, the uncertain preventive benefit of cerclage, potential operative complications, and the need for cesarean delivery. Following shared decision‐making, written informed consent was obtained.

**FIGURE 2:**
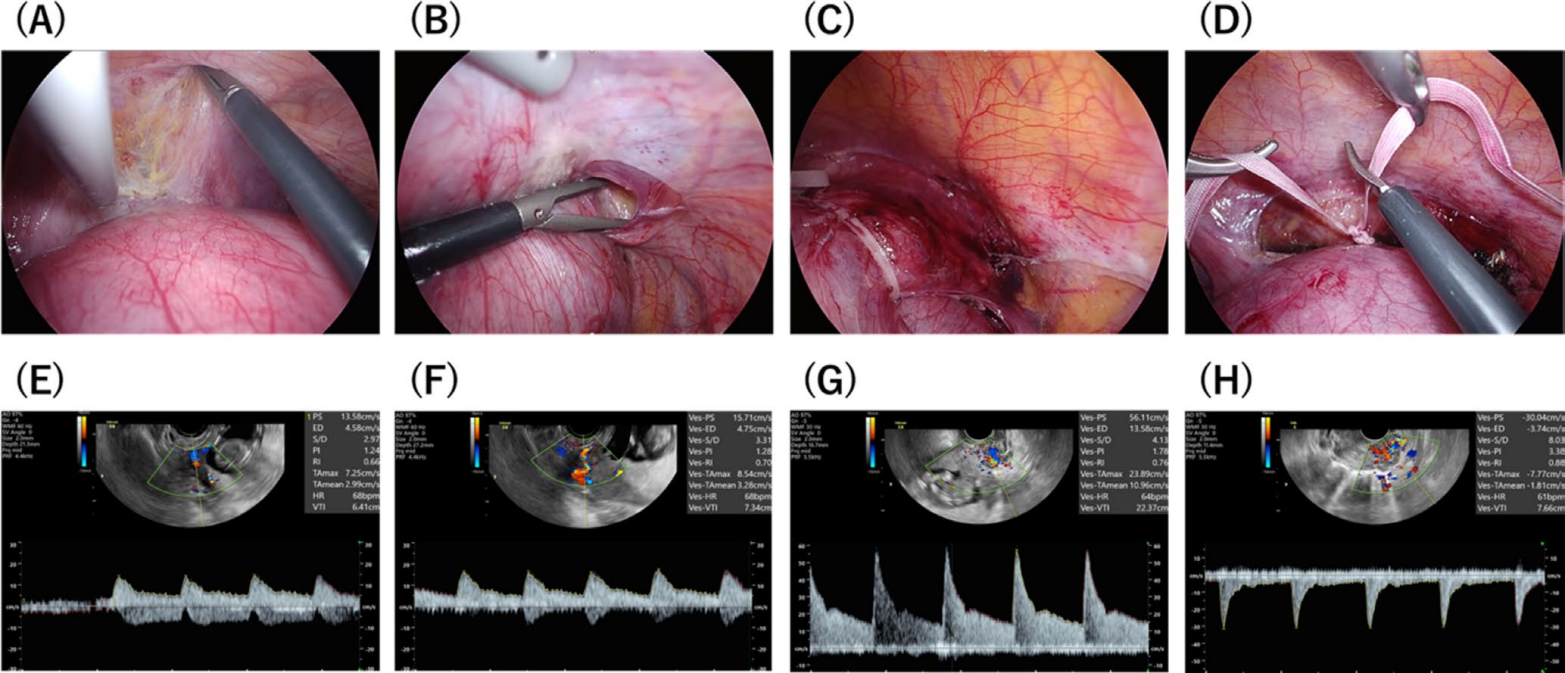
Intraoperative findings and uterine artery Doppler assessment during laparoscopic transabdominal cervicoisthmic cerclage at 13 weeks of gestation. (A) Incision of the vesicouterine peritoneum. (B) Opening of the retroperitoneal space anterior to the ureter. (C) Following temporary tightening of the tape, transvaginal ultrasonography was performed to confirm the level of cerclage placement and assess bilateral uterine artery flow. (D) The tape was secured after confirming preservation of bilateral uterine artery flow. (E, F) Preoperative Doppler waveforms of the left and right ascending uterine arteries. (G, H) Corresponding post‐cerclage Doppler waveforms demonstrating preserved antegrade flow in both ascending uterine arteries.

At 13 weeks and 3 days of gestation, a modified laparoscopic transabdominal cervicoisthmic cerclage was performed according to the technique described by Shin et al. [5] (Figure 2A–D). The vesicouterine peritoneum was incised, and the retroperitoneum was opened anterior to each ureter. Teflon tape was passed around the uterine isthmus lateral to the ascending branches of the uterine arteries. Blood flow in the bilateral ascending branches of the uterine arteries was assessed using transvaginal Doppler ultrasonography, and the tape was secured after confirming the absence of flow interruption or reversal (Figure 2E–H). No intraoperative complications occurred, and the patient was discharged on postoperative day 5.

**FIGURE 3:**
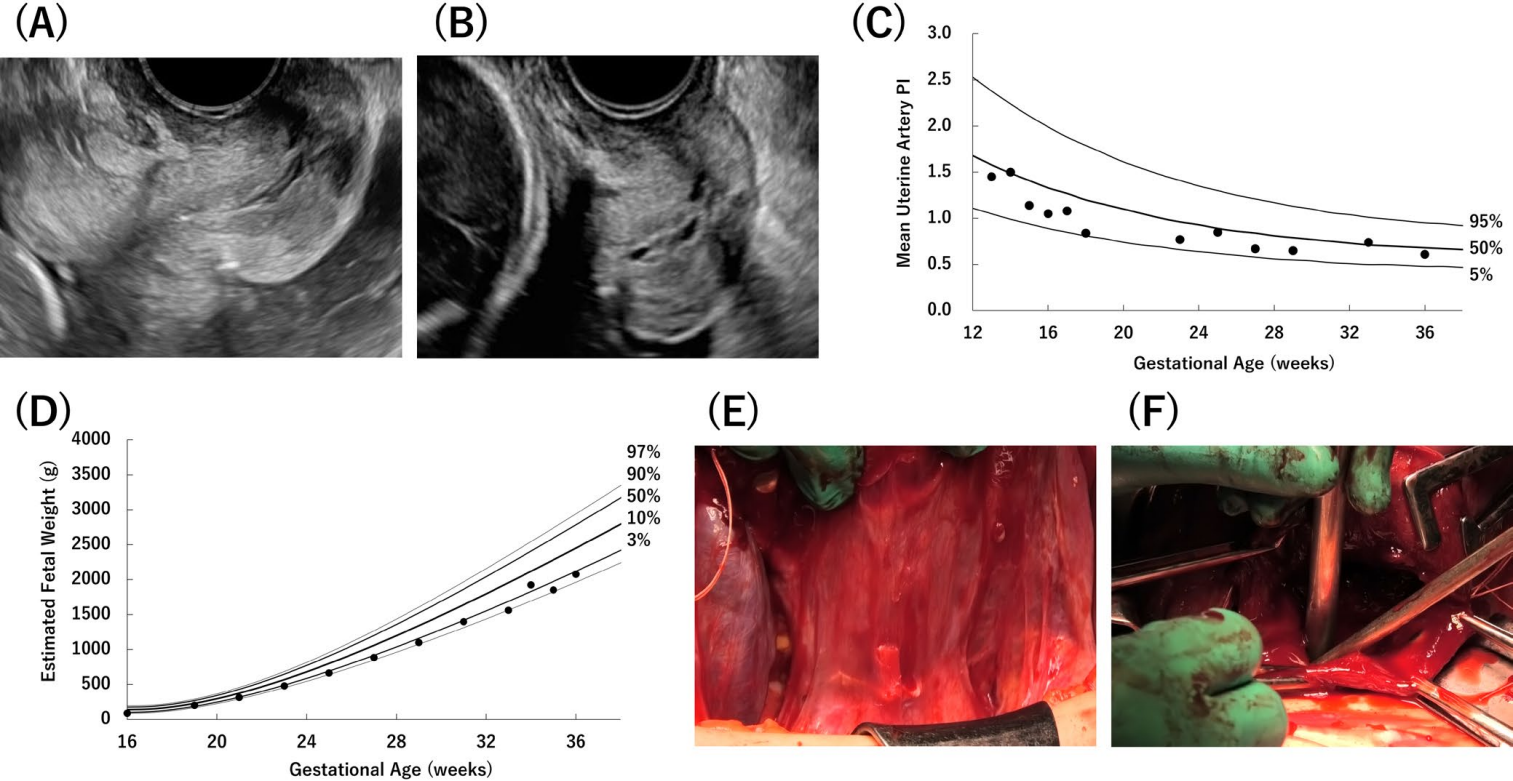
Postoperative cervical morphology, uterine artery Doppler findings, fetal growth, and intraoperative findings at cesarean delivery. (A, B) Serial transvaginal ultrasonographic images of the cervix obtained (A) on postoperative day 1 at 13 weeks of gestation and (B) at 21 weeks of gestation. (C) Serial mean uterine artery pulsatility index measurements plotted against gestational age. Solid curves represent the 5th, 50th, and 95th percentiles of the reference range. (D) Serial estimated fetal weight measurements plotted against gestational age. Solid curves represent the 3rd, 10th, 50th, 90th, and 97th percentiles of the Japanese reference range; black circles indicate measurements from the present patient. (E, F) Intraoperative findings at cesarean delivery: (E) after delivery of the infant, the Teflon tape was identified at the uterine isthmus beneath filmy adhesions, and (F) cervical dilation was performed from the uterine cavity using cervical dilators measuring 8–15 mm in diameter.

The cervical morphology remained stable throughout pregnancy (Figure 3A,B). The mean uterine artery pulsatility index remained within the gestational age‐specific reference range [6] (Figure 3C). The estimated fetal weight remained between the 3rd and 10th percentiles of the Japanese reference standard without an apparent decline in growth velocity [7] (Figure 3D). No hypertensive disorders of pregnancy, gestational diabetes, or prelabor membrane rupture occurred.

A planned cesarean delivery was performed at 37 weeks and 5 days of gestation. The operative time was 60 min, and the estimated blood loss, including amniotic fluid, was 390 g. A 2114‐g infant, classified as small for gestational age, was delivered through a low transverse uterine incision. The Apgar scores were 8 and 9 at 1 and 5 min, respectively, and the umbilical artery pH was 7.259.

After delivery, the Teflon tape was removed because of severe cervical stenosis and the anticipated risk of impaired menstrual outflow. The cervix was dilated from the uterine cavity using cervical dilators (Figure 3E,F). The postoperative courses for both the mother and infant were uneventful.

## 3 Discussion

This case report illustrates an extreme form of acquired post‐conization cervical injury characterized by near‐complete loss of the vaginal cervix, external os obliteration, hematometra, technically challenging recanalization, and dependence on repeated cervical dilation. These preconception findings indicated that the cervix was not merely shortened but profoundly disrupted both anatomically and functionally. The 11.9‐mm residual endocervical glandular length represented only one component of this broader phenotype.

Although the patient had no history of miscarriage or preterm birth, the residual anatomy raised concerns regarding its ability to provide adequate cervical support throughout pregnancy, whereas the near‐complete loss of the vaginal cervix rendered transvaginal cerclage technically unfeasible. Laparoscopic transabdominal cervicoisthmic cerclage was therefore selected after considering the anatomical findings, alternative management options, operative risks, and patient's preferences.

Conventional history‐based indications for transabdominal cerclage may not adequately address profound acquired cervical anatomical failure, which should be distinguished from uncomplicated post‐conization or sonographic cervical shortening.

Cerclage is classified as history‐indicated, ultrasound‐indicated, or physical examination‐indicated. In singleton pregnancies with a previous spontaneous preterm birth and a short cervix in the second trimester, cerclage reduces the risk of recurrent preterm birth [8]. By contrast, a consistent benefit has not been demonstrated in women without a previous spontaneous preterm birth and a cervical length of < 25 mm, although a benefit has been suggested when the cervical length is < 10 mm [9]. In this patient, the 11.9‐mm measurement represented the distance from the external cervical os to the cranial end of the residual endocervical glandular region rather than the conventional sonographic cervical length measured between the internal and external cervical os. Accordingly, it cannot be interpreted using established thresholds for ultrasound‐indicated cerclage. More importantly, the abnormality was clinically evident before pregnancy, as demonstrated by hematometra, loss of an identifiable external os, failed transvaginal drainage, technically challenging recanalization, and dependence on repeated cervical dilation. The key clinical question was therefore not whether the cervix was conventionally “short,” but whether the markedly disrupted residual cervical anatomy could provide sufficient mechanical support throughout pregnancy.

Pregnancy after cervical conization is associated with an increased risk of second‐trimester miscarriage, preterm birth, preterm prelabor rupture of membranes, and chorioamnionitis; this risk increases after deeper or repeated excisions [2, 3, 4, 10]. Nevertheless, routine prophylactic cerclage after conization has not demonstrated a clear benefit and has been associated with an increased risk of preterm birth in some studies [11, 12, 13]. These findings do not support cerclage based solely on a history of conization. Therefore, the present patient should not be regarded as representative of women undergoing routine prophylactic cerclage after repeated cervical conization. Rather than uncomplicated post‐conization cervical shortening, she exhibited near‐complete loss of the vaginal cervix, hematometra caused by obliteration of the external cervical os, and a surgically reconstructed cervical canal requiring repeated dilation to maintain patency. This presentation represents the extreme end of the spectrum of acquired post‐conization cervical injury, in which conventional transvaginal cerclage is technically unfeasible. Therefore, such patients should be distinguished from the broader population of women with a history of cervical conization when considering cerclage indications.

Severe cervical stenosis may cause hematometra and dysmenorrhea and may contribute to infertility by obstructing sperm passage or making catheter placement for intrauterine insemination or embryo transfer difficult [14]. In the present patient, pregnancy was achieved only after cervical reconstruction, repeated cervical dilation, and intrauterine insemination. Such patients may experience substantial physical, psychological, temporal, and financial burdens before conception. For those requiring fertility treatment and who may have limited opportunities for future pregnancy, an indication framework that relies solely on pregnancy loss or a history of miscarriage or preterm birth may not adequately address severe acquired cervical anatomical failure.

Nevertheless, limited reproductive opportunities and a strong desire to avoid pregnancy loss do not, by themselves, justify invasive surgery. Severe acquired cervical anatomical failure and the technical infeasibility of transvaginal cerclage should remain the principal indications for intervention, with the course to conception and the patient's values incorporated through shared decision‐making.

The modified laparoscopic technique described by Shin et al. [5] was used, in which the tape was placed lateral to the uterine arteries without direct arterial dissection. This approach may reduce the risk of vascular injury; however, as the uterine isthmus is encircled by the uterine vessels, its potential effects on uteroplacental perfusion and fetal growth remain a concern. Seo et al. reported favorable surgical and pregnancy outcomes in 30 patients without evident adverse effects on uterine artery flow or fetal growth [15]. In the present patient, the mean uterine artery pulsatility index remained within the gestational age‐specific reference range throughout pregnancy, suggesting preserved uterine artery perfusion.

Nevertheless, the infant was small for gestational age, weighing 2114 g at 37 weeks and 5 days of gestation. The estimated fetal weight remained between the 3rd and 10th percentiles throughout pregnancy without an apparent decline in growth velocity. Both parents were of relatively short stature (maternal height, 148 cm; paternal height, 167 cm), and the maternal pre‐pregnancy weight was 39 kg, which may have contributed to the infant's small size. However, a normal uterine artery pulsatility index does not exclude a possible effect of cerclage on fetal growth. Further studies are required to evaluate uteroplacental perfusion and fetal growth following this modified technique.

This report describes a single patient without comparison with expectant management; therefore, the favorable pregnancy outcome cannot establish the necessity or effectiveness of surgery. The infant was small for gestational age, and a possible effect of cerclage on fetal growth could not be excluded. The indications for transabdominal cerclage after repeated conization in patients without prior miscarriage or preterm birth remain undefined. However, when profound acquired cervical anatomical failure renders transvaginal cerclage technically unfeasible, individualized decision‐making should consider the anatomical findings, alternative management options, operative risks, and patient's values.

Laparoscopic transabdominal cervicoisthmic cerclage was performed during pregnancy in a patient with profound cervical tissue loss and distortion after repeated conization, in whom transvaginal cerclage was technically unfeasible. Although the effectiveness of this approach cannot be established from a single patient, profound anatomical cervical defects may constitute a distinct clinical scenario beyond the scope of conventional history‐based indications for cerclage.

Written informed consent for the publication of this case report and the accompanying images was obtained from the patient.

## Author Contributions

**Takayuki Iriyama:** conceptualization, investigation, supervision, writing – review and editing. **Gentaro Izumi:** investigation, writing – review and editing, supervision. **Osamu Hiraike:** investigation, supervision, writing – review and editing. **Miyuki Harada:** supervision, project administration, writing – review and editing. **Yasushi Hirota:** supervision, project administration, writing – review and editing. **Kensuke Suzuki:** conceptualization, data curation, investigation, visualization, writing – original draft.

## Disclosure

This article has not been previously presented at any conference or scientific meeting.

## Ethics Statement

The authors have nothing to report.

## Consent

Informed consent was received from the patient.

## Conflicts of Interest

The authors declare no conflicts of interest.

## Data Availability

The clinical data supporting the findings of this case report are not publicly available due to patient privacy and ethical restrictions. Because this report concerns a rare case and includes detailed individual‐level clinical information and medical images, there remains a risk of indirect patient identification even after de‐identification. Therefore, the data have not been deposited in a public repository. Limited de‐identified data may be made available by the corresponding author upon reasonable request, subject to appropriate institutional and ethical approval.
